# Phylogenomic resolution of Imparidentia (Mollusca: Bivalvia) diversification through mitochondrial genomes

**DOI:** 10.1007/s42995-023-00178-x

**Published:** 2023-06-19

**Authors:** Yu Wang, Yi Yang, Lingfeng Kong, Takenori Sasaki, Qi Li

**Affiliations:** 1 Key Laboratory of Mariculture, Ministry of Education, Ocean University of China, Qingdao, 266003 China; 2 Laboratory for Marine Fisheries Science and Food Production Processes, Laoshan Laboratory, Qingdao, 266237 China; 3 The University Museum, The University of Tokyo, Tokyo, 113-0033 Japan

**Keywords:** Imparidentia, Phylogeny, Mitochondrial genome

## Abstract

Despite significant advances in the phylogenomics of bivalves over the past decade, the higher-level phylogeny of Imparidentia (a superorder of Heterodonta) remains elusive. Here, a total of five new mitochondrial sequences (*Chama asperella*, *Chama limbula*, *Chama dunkeri*, *Barnea manilensis* and *Ctena divergens*) was added to provide resolution in nodes that required additional study*.* Although the monophyly of Lucinida remains less clear, the results revealed the overall backbone of the Imparidentia tree and the monophyly of Imparidentia. Likewise, most relationships among the five major Imparidentia lineages—Lucinida, Cardiida, Adapedonta, Myida and Venerida—were addressed with a well-supported topology. Basal relationships of Imparidentia recovered Lucinidae as the sister group to all remaining imparidentian taxa. Thyasiridae is a sister group to other imparidentian bivalves (except Lucinidae species) which is split into Cardiida, Adapedonta and the divergent clade of Neoheterodontei. Neoheterodontei was comprised of Venerida and Myida, the former of which now also contains Chamidae as the sister group to all the remaining venerid taxa. Moreover, molecular divergence times were inferred by calibrating nine nodes in the Imparidentia tree of life by extinct taxa. The origin of these major clades ranged from Ordovician to Permian with the diversification through the Palaeozoic to Mesozoic. Overall, the results obtained in this study demonstrate a better-resolved Imparidentia phylogeny based on mitochondrial genomes.

**Supplementary Information:**

The online version contains supplementary material available at 10.1007/s42995-023-00178-x.

## Introduction

Euheterodonta is accomplished by two major clades (Anomalodesmata and Imparidentia) and is recognized as the most diverse group of Bivalvia (MolluscaBase 2021). Imparidentia is a diverse clade of marine, brackish and freshwater bivalve molluscs that was established by Bieler et al. (2014) and includes well-known and economically important groups such as shipworms, giant clams, geoduck clams and cockles. Imparidentia taxa are quite variable in their habits which include burrowing in sediments, attaching to intertidal reefs, boring into rock and wood, or living with other invertebrates. Therefore, Imparidentia adopts a multitude of different alimentation modes including filter feeding, algal photosymbiosis and bacterial chemosymbiosis (Campbell and Bottjer 1995; Li et al. 2018). Besides their considerable ecological importance in community structure, numerous Imparidentia species could be regarded as trophic resources. Thus, many efforts have been made to understand the phylogeny of the Imparidentia using shell morphology, anatomy and available molecular markers (Bieler et al. 2014; Lemer et al. 2019).

The internal structure of the group remained opaque to zootomic, mitochondrial, nuclear fragment-based or other approaches even though some phylogenetic patterns of Imparidentia have been identified. This group clearly contains the order Lucinida, Cardiida, Adapedonta, and the large Neoheterodontei clade (first defined by Taylor et al. 2007) and was composed of the orders Myida and Venerida and some families whose systematic position is uncertain (Bieler et al. 2014) although Lemer et al. (2019) subsequently determined the positions of some of these families. However, previous phylogenetic studies showed low resolution of deeply dividing lineages within the Imparidentia such as Adapedonta (Lemer et al. 2019). In addition, it is unclear whether Thyasiridae is included in Lucinda and the relationship between Lucinidae and Thyasiridae was poorly resolved in a previous analysis (Bieler et al. 2014). Furthermore, the systematic positions of a large number of families within Imparidentia could not be determined with certainty, for example, the position of the extremely long-branched Chamidae was conflictive in previous studies (González et al. 2015; Taylor et al. 2007). Pholadidae is a family of the order Myida adapted to boring into a range of substrates, however, there is no definitive conclusion as to when this habit evolved.

Mitochondrial genomes, which are characterized by their small size, maternal inheritance, rare recombination and high evolutionary rate (Boore 1999; Cameron 2014; Curole and Kocher 1999), provide a suitable data source for phylogenetic inference. Typical metazoan mitochondrial DNA (mtDNA) is a compact circular molecule that comprises 13 protein-coding genes, two rRNA genes and 22 tRNAs used for translation within the organelle (Boore 1999; Breton et al. 2014). Mitochondrial genomes have higher mutation rates and smaller effective population sizes (Ne) than nuclear DNA (Cameron 2014; Curole and Kocher 1999; Eberle et al. 2020) and thus could provide more phylogenetic information compared with nuclear or mitochondrial gene fragments. Multiple levels of phylogenetic signal, such as nucleotide, amino acid, gene arrangement pattern, small subunit ribosomal RNA (SSU rRNA) mitogenome architecture and RNA secondary structure, have been used for analysising (Dowton and Austin 1999; Liu et al. 2022; Mikkelsen et al. 2018; Salvi and Mariottini 2012; Shao et al. 2001; Wu et al. 2020). Denser and strategic taxonomic sampling of mitogenomes for mitogenome characterization has been implemented by next-generation sequencing methods (Smith 2016). Complete sequences of mitochondrial genomes of Imparidentia bivalves have been determined to assess the variability of their gene contents and features of the genome organisation (Dreyer and Steiner 2006). The evolution of gene rearrangements and phylogenetic implications have been informed using the mitogenomes of Tellinoidea and Solenoidea (Sun et al. 2020; Yuan et al. 2012). A subsequent study based on mitogenomes provided much-expanded taxon sampling for the family Veneridae (Bivalvia) and explored the evolutionary relationships among subfamilies of Veneridae (Wang et al. 2021). Such enriched diversity and divergence in Imparidentia mitogenomes, for which many genomic resources are available, provide an opportunity to derive a better understanding of the internal phylogenetic inference within Imparidentia and the evolution of mitogenomes.

In the current study, five mitochondrial genomes of Imparidentia (*Ctena divergens*, *Barnea manilensis*, *Chama asperella*, *Chama limbula*, and *Chama dunkeri*) were newly sequenced to reconstruct the phylogenetic relationship of the superorder Imparidentia. In addition, a comparative analysis of 86 Imparidentia mitogenomes was probed. Our aims are: (1) to improve the phylogenetic resolution within Imparidentia; and (2) to date the main cladogenetic events during the diversification of the superorder Imparidentia.

## Results

### Mitochondrial genome organization

**Fig. 1:**
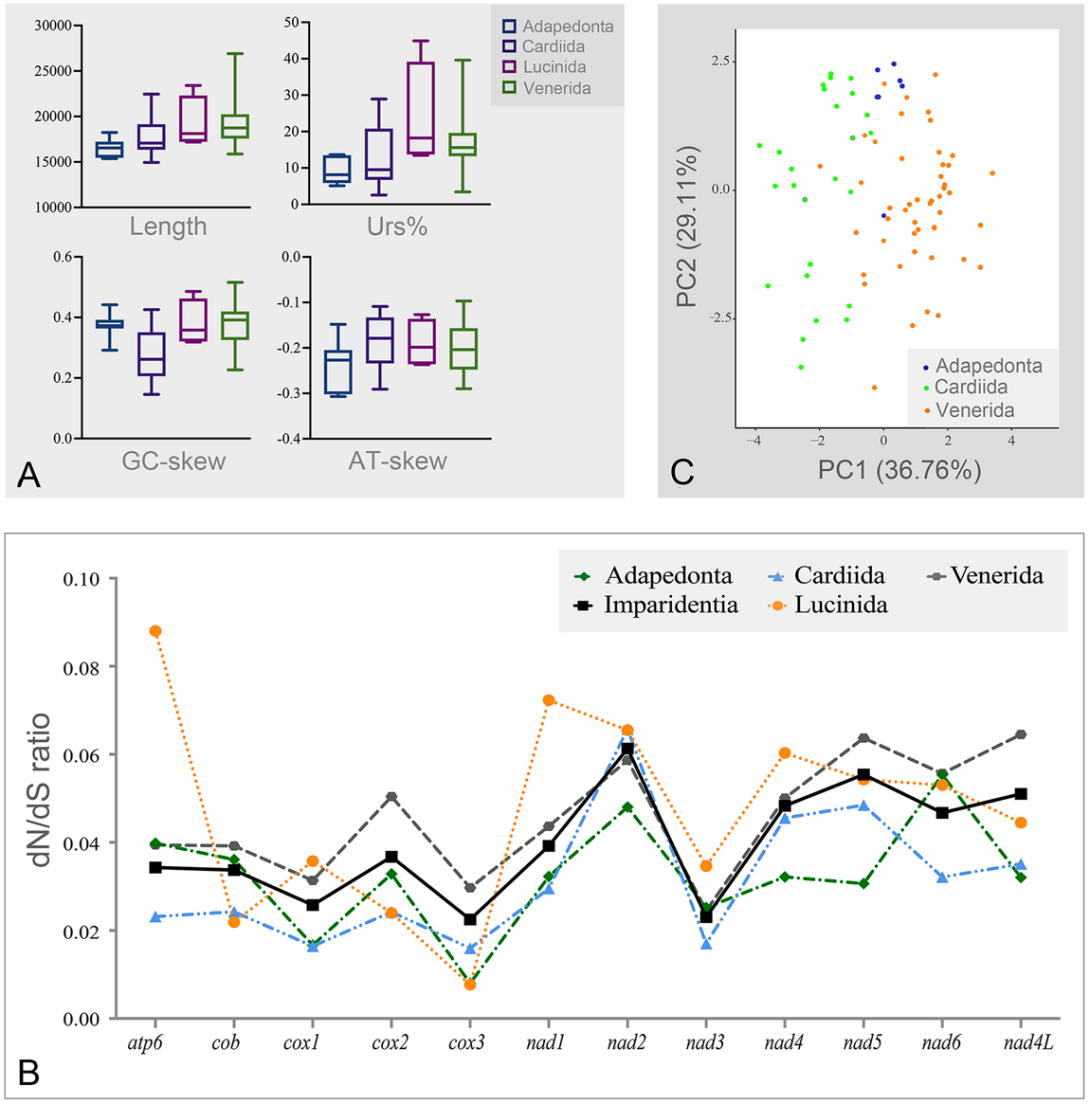
The futures of Imparidentia bivalves mt genmones. **A** Five features of mitochondrial genomes compared across four Imparidentia orders. Urs%, median percentage of untranslated regions; Length: the length of the complete mitochondrial genome. **B** The ratio of synonymous nucleotide substitutions (dN)/non-synonymous nucleotide substitutions (dS) were calculated. Input data are shown in Supplementary Table S2. **C** Imparidentia mitogenomics PCA. Input data are shown in Supplementary Table S1. Different orders are indicated with different colors. *PC* principal component

The complete mitochondrial genomes of *C. asperella*, *C. dunkeri*, *C. divergens* and *B. manilensis,* and the nearly complete mitochondrial genome of *C. limbula* (only lacking two tRNAs), were obtained and deposited in GenBank with accession number MZ701706, MZ557447, MZ540208, MZ701705, and MZ688407, respectively. They were analyzed together with an additional 81 mitochondrial genomes of Imparidentia downloaded from GenBank (for details and GenBank accession numbers, see supplementary Table S1). The complete mitochondrial genomes (mtDNAs) of these 86 bivalves varied in size from 14,979 bp (*Cerastoderma edule*) to 26,915 bp (*C. asperella*). The size of putatively unstranslated regions (Urs) spanned from < 1% (*Villorita cyprinoides*) to > 39% (Chamidae). The number of annotated genes was more stable, ranging from 33 (*Sinonovacula constricta*) to 42 (*Fulvia mutica*). As summarized in Fig. 1A, the median of URs (%) increased in the order: Cardiida > Lucinida > Venerida > Adapedonta, and in terms of mtDNA length: Venerida > Lucinida > Cardiida > Adapedonta. In addition, these orders differed significantly in terms of their A-T and G-C skews. Finally, the dN/dS ratio for each protein-coding gene (PCG) among the 86 mitochondrial genomes is given in Fig. 1B. The highest value was in *nad2* (dN/ dS = 0.061), while the lowest was in *cox3* (dN/dS = 0.022) (Supplementary Table S2).

### Summarizing data: principal component analysis (PCA)

All Imparidentia bivalve (except two species of Myida and four species of Lucinida) mitogenomic features are listed in Supplementary Table S1. The results of the principal component analysis (PCA) are given in Fig. 1C and Supplementary Fig. S1. The first two principal compnents account for 65.87% of the matrix data set variance. The larger taxonomic combinations are easy to identify: Adapedonta was at the top, Cardiida was on the left side and the wide cluster of Venerida was on the right side (Fig. 1C). The family Veneridae almost formed a single cluster, contrastingly, pointes of some families are sharply separated from other species belonging to the same family, e.g., Vesicomyidae and Chamidae. The superfamily Tellinoidea (except family Tellinidae) clustered into two groups: one represented by Donacidae, the other represented by Psammobiidae, Semelidae, Solecurtidae, and Scrobiculariidae (Supplementary Fig. S1).

### Phylogenetic relationships

**Fig. 2:**
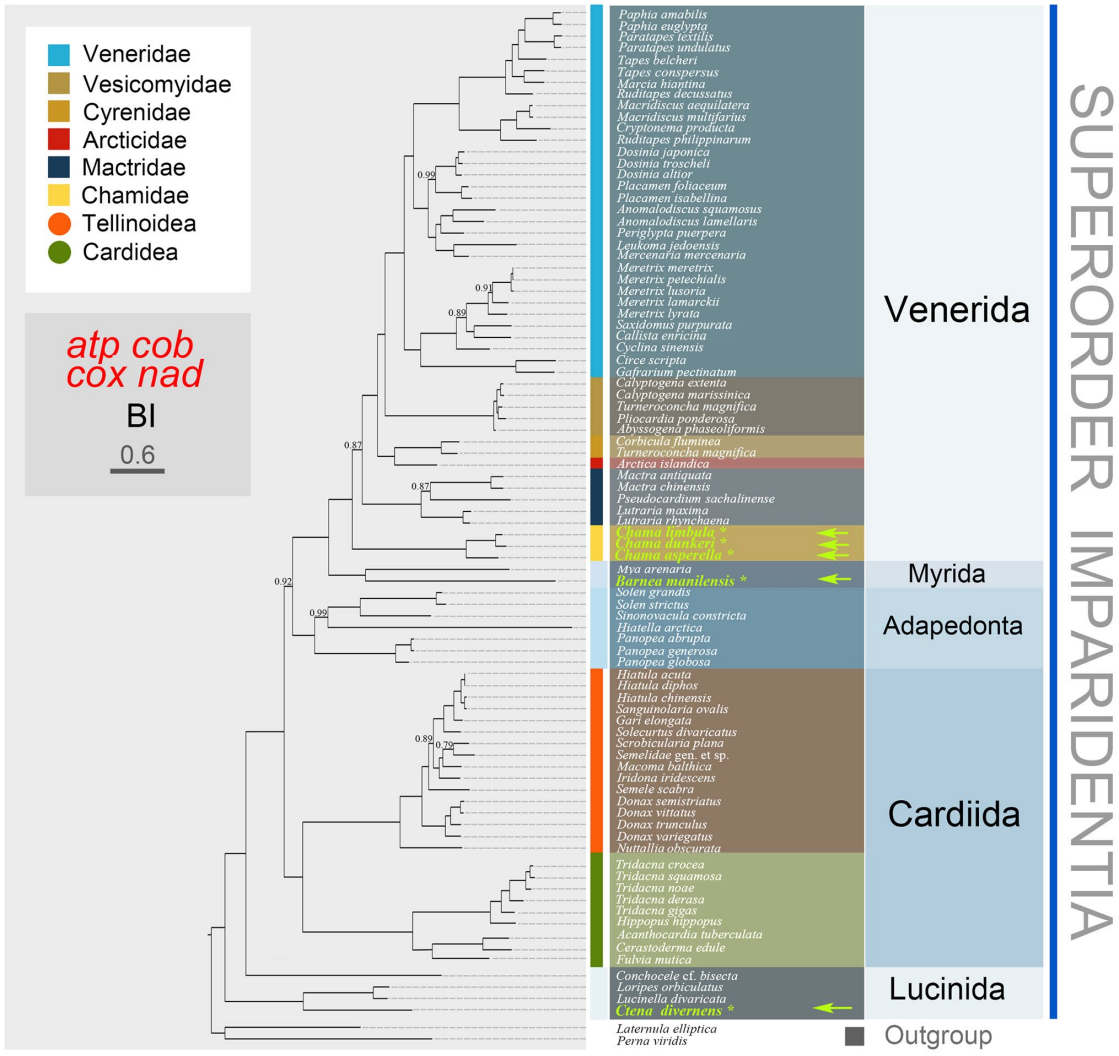
Phylogenetic tree of Imparidentia inferred from Bayesian inference analyses based on mt genomes. Bayesian posterior probability is shown in the branches. The newly sequenced mt genomes are indicated with an asterisk (*). Nodes with no labels were maximally supported (PP = 1.00)

Phylogenetic trees based on an amino acid matrix data set (containing 2998 sites) deduced from 12 PCGs (except *atp8* gene due to many uncertainties in annotation and orthology) were inferred using BI and ML methods. The BI tree (Fig. 2) and ML phylogram (Supplementary Fig. S2) had analogical topologies but with some differences. Lucinida occupied the basal position clustering in a well-supported clade with Cardiidae, Adapedonta, Myida and Venerida. Thyasiridae, however, was a stable sister group to all the remaining Imparidentia instead of forming a clade with other lucinida taxa. Cardiida was subdivided into two clades, Cardioidea and Tellinoidea. The earliest diverging lineage of Cardioidea was Cardiidae which was divided into two subclades with full support. *Nuttallia obscurata* (Psammobiidae) occupied the basal position within the Tellinoidea clade, rather than clustered with other Psammobiidae taxa. Solecurtidae and Psammobiidae formed a well-supported clade. Members of the families Semelidae and Tellinidae grouped together in a maximally supported clade (100% PP) within the superfamily Tellinoidea. It is noteworthy that *Iridona iridescens* (Tellinidae) was a sister group to the *Macoma balthica* + Semelidae clade with well support both in the ML tree (Supplementary Fig. S2) and BI tree (Fig. 2), but the *M. balthica* + Semelidae clade was with poor support in the ML tree. Adapedonta, consisting of Solenidae, Hiatellidae and Pharidae, was resolved as a sister to the well-supported Myida + Venerida clade with weak support in the ML tree, but with maximal support in the BI tree. Within the Adapedonta, *Hiatella* is separated on a long branch from the two *Solen* species and *Sinonovacula constricta*, although with weak support. *Panopea* was a sister group to the *Hiatella* + *Solen* + *Sinonovacula* clade. Within the Myida branch, *Barnea manilensis* was sister to *Mya arenaria.* Myida was sister to Venerida with Adapedonta being their most immediate outgroup. The evolutionary relationship within Venerida shows more uncertainty, even after the addition of Chamoidea. Chamoidea occupied the basal position within Venerida. *Pseudocardium sachalinense* and *Lutraria* formed a weakly supported clade in the ML tree, however, *P. sachalinense* was sister to *Mactra* in the BI tree (PP = 0.87) within the Mactridae. Within Veneridae, Callistinae and Meretricinae formed a strongly supported clade that was sister to Cyclininae presented by *Cyclina sinensis* (PP = 1.00) in the BI tree, whereas the Callistinae + Cyclininae clade was sister to the Meretricinae with moderately supported (bootstrap support, BS = 73) in ML analysis.

### Molecular dating of Imparidentia reveals ancient diversification

**Fig. 3:**
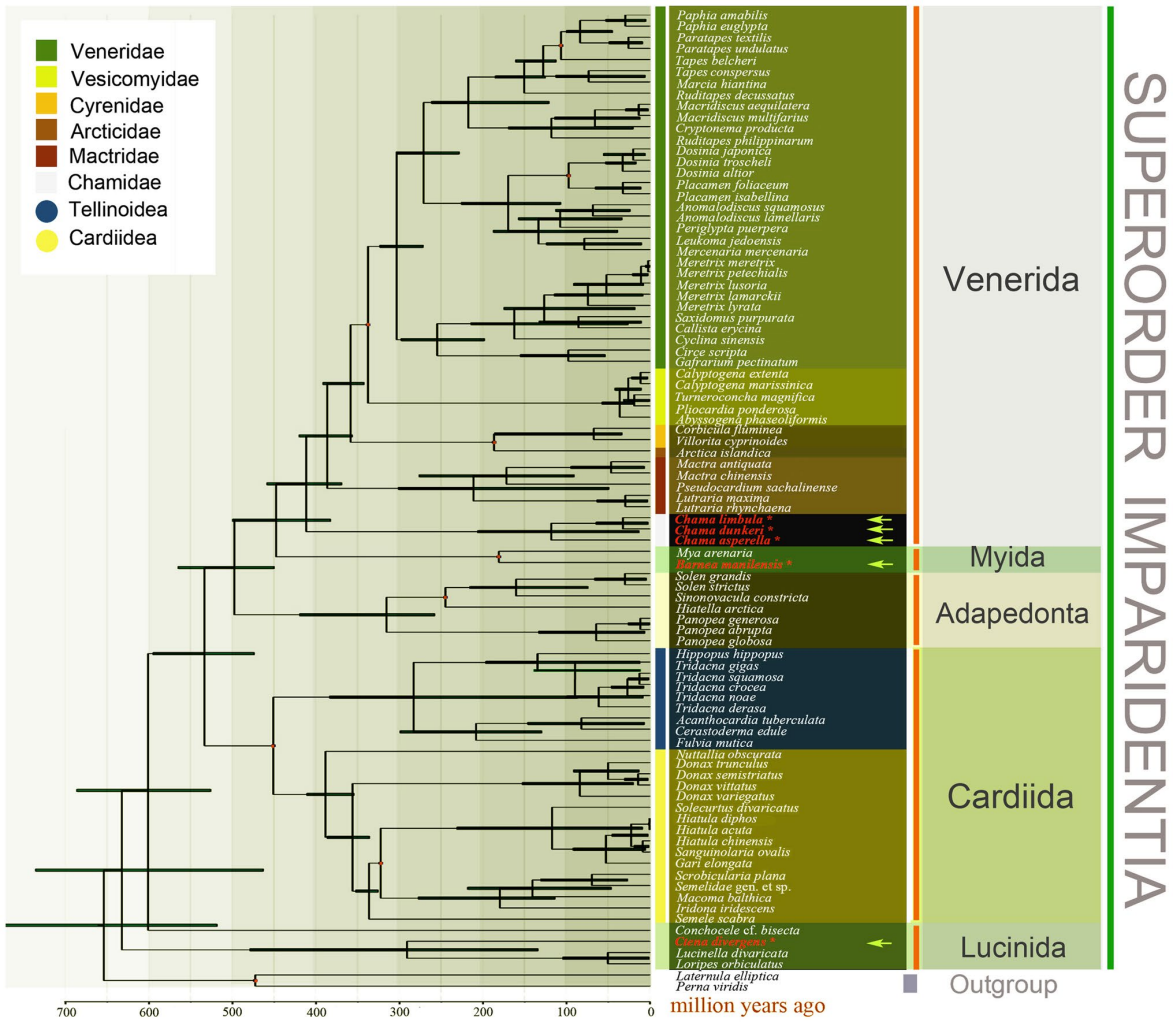
Divergence time estimation analysis of Imparidentia derived via Bayesian inference with 95% highest posterior density interval (HPD) reported as bold bars. Fossil samples used as prior are marked by red circles. The newly sequenced mt genomes are indicated with asterisks and arrows

Divergence times of clades were performed using nine fossil calibrations (Fig. 3). Dating performed under a relaxed molecular clock model recovered a Precambrian age for the crown group of Imparidentia, i.e., 632 Mya (million years ago) [Precambrian]. Diversification dates for major clades of Imparidentia were deduced as follows: Lucinida: 387 Mya; Cardiida: 450.76 Mya (highest posterior density interval (HPD): 449–594 Mya); Adapedonta: 315.1 Mya (HPD: 258–419 Mya); Myoida: 181 Mya (HPD: 179–183 Mya); Venerida: 411 Mya (HPD: 382–499 Mya); Neoheterodontei: 497 Mya (HPD: 450–565 Mya). Dates for all other nodes are shown in Supplementary Fig. S3.

## Discussion

### Mitochondrial evolution

Although the 12 protein-coding genes had various dN/dS ratios among several orders, all dN/dS ratios of Imparidentia bivalves obtained in this work were less than 1, indicating that they were all subjected to purifying selection. Overall, *nad* genes had more non-synonymous sites than *cox* genes and *cob* genes. This trend is consistent with the hypothesis that genes encoding subunits of cytochrome c oxidase and cytochrome b are more conserved than NADH dehydrogenase genes (Saccone et al. 1999). It is noteworthy that the *nad2* gene had the highest dN/dS value, indicating that this gene has been subjected to the loosest purifying selection. Furthermore, compared with other PCGs (protein-coding genes), the *nad2* gene has accumulated more non-synonymous mutations.

The results of the PCA showed that Cardiida was separated from Venerida on PC1 (36.76%), whereas some Venerida taxa clusted with Cardiida (Fig. 1C). Although Adapedonta species gather as a group, Adapedonta could not be distinguished from the remaining two orders due to the small sample size. Further studies applying more species and more features of the mitochondrial genome are required to better comprehend the factors affecting the mt genome in bivalves.

### Phylogenetic analysis

We reconstructed the phylogeny of Imparidentia bivalves using amino acid sequences of the 12 mitochondrial protein-coding genes and investigated the evolutionary patterns of Imparidentia mitochondrial genome. The BI analysis provided a remarkably well-supported and well-resolved phylogeny of the group, whereas in the ML analysis some higher-level clades of Imparidentia were only weakly supported.

The monophyly of the much-discussed clade Lucinida was rejected in the present study, which is consistent with the results of previous analyses (Taylor and Glover 2006; Taylor et al. 2007). Moreover, our analyses failed to support prior hypotheses that Thyasiridae and Lucinidae occupy the basal position of Imparidentia (Bieler et al. 2014; Combosch et al. 2017a). Nevertheless, the finding that Thyasiridae is the sister group to the rest of Imparidentia with Lucinidae excluded was strongly supported in both the ML and BI trees. Thus, the position of Thyasiridae in our analysis confirmed one of the two possibilities proposed by Lemer et al. (2019).

Cardiida formed a stable branch consisting of Cardioidea + Tellinoidea in both the BI and ML trees, albeit with variable support, which is consistent with previous studies (Bieler et al. 2014; Taylor et al. 2007; Williams et al. 2017). The relationship among these families of the superfamily Tellinoidea remains poorly resolved as reported previously (Sun et al. 2020; Yuan et al. 2012). Therefore, further phylogenetic studies including more taxa of the superfamily Tellinoidea are required.

The position of Adapedonta was inconclusive in previous analyses. Lemer et al. (2019) found that Adapedonta is the sister group to Cardiida, whereas Adapedonta is the sister group to Myida and Venerida in our analysis, which is consistent with previous analyses using mitochondrial genomes (Williams et al. 2017). Adapedonta was a well-supported clade comprising two superfamilies Hiatelloidea and Solenoidea (= Solenidae + Pharidae) in both the BI and ML trees (Fig. 2; Supplementary Fig. S2), and this grouping has been recognized in previous phylogenetic studies (Bieler et al. 2014; Taylor et al. 2007, 2009; Yuan et al. 2012). Nevertheless, this result conflicts with previous findings based on morphology which suggest that the Hiatelloidea was a member of the Myida (Newell 1965). However, there is no strong morphological apomorphy that unites these two taxa. In the current mtDNA phylogenetic analyses, *Hiatella* is separated on a long branch from the three Solenoidea species (Fig. 2; Supplementary Fig. S2), whereas in some previous molecular analyses *Hiatella* grouped with Panopea (Bieler et al. 2014; Taylor et al. 2007).

A putative clade, named Neoheterodontei by Taylor et al. (2007), including Myida and an array of families that we allocate to a redefined Venerida (Fig. 2), has been reported in other molecular phylogenies (Bieler et al. 2014; González et al. 2015; Lemer et al. 2019). The high-level taxonomic ranks of Neoheterodontei were likewise stable and highly supported, except Mactroidea formed a cluster to all other veneridans (exclude Chamidae taxa) with low support in the ML tree. Venerida was less clearly resolved, the placements of Chamoidea, Mactroidea, Arcticoidea, and Veneroidea still being problematic. The position of Chamidae has been long discussed (Combosch et al. 2017a; González et al. 2015; Taylor et al. 2007) but was well resolved as a member of Venerida in present analyses with full support. Chamaidae was the sister group to the rest of the Venerida taxa in this study. In previously published gene trees, Chamidae grouped with Hemidonacidae, Glossidae, Trapezidae, Cardiidae and Veneridae (Bieler et al. 2014; Combosch et al. 2017a; Lemer et al. 2019), so its definitive position is less clear. Furthermore, Arcticidae was sister group either to Vesicomyidae + Veneridae or to Trapezidae in previous studies (Bouchet et al. 2010; Lemer et al. 2019), but was closely related to Cyrenidae within a well-supported clade in the current study. Mactroidea represented by Mactridae was sister group to the rest of Neoheterodontei, excluding Chamoidea and Myida, which conflicted with a published hypothesis (Taylor et al. 2007). Vesicomyidae clustered with Veneridae rather than with Glossidae, which is in contrast to the findings of Bouchet et al. (2010). The evolutionary relationship of Veneridae has been investigated and contested in previous studies (Chen et al. 2011; Kappner and Bieler 2006; Mikkelsen et al. 2006; Wang et al. 2021). Veneridae is generally considered a complicated group with two subclades as reported here. However, relationships among venerid lineages differed slightly in the BI and ML trees (Fig. 2; Supplementary Fig. S2). Investigations of other nominal venerid taxa will be necessary to fully resolve the phylogeny of Veneridae.

### Divergence time estimation

**Fig. 4:**
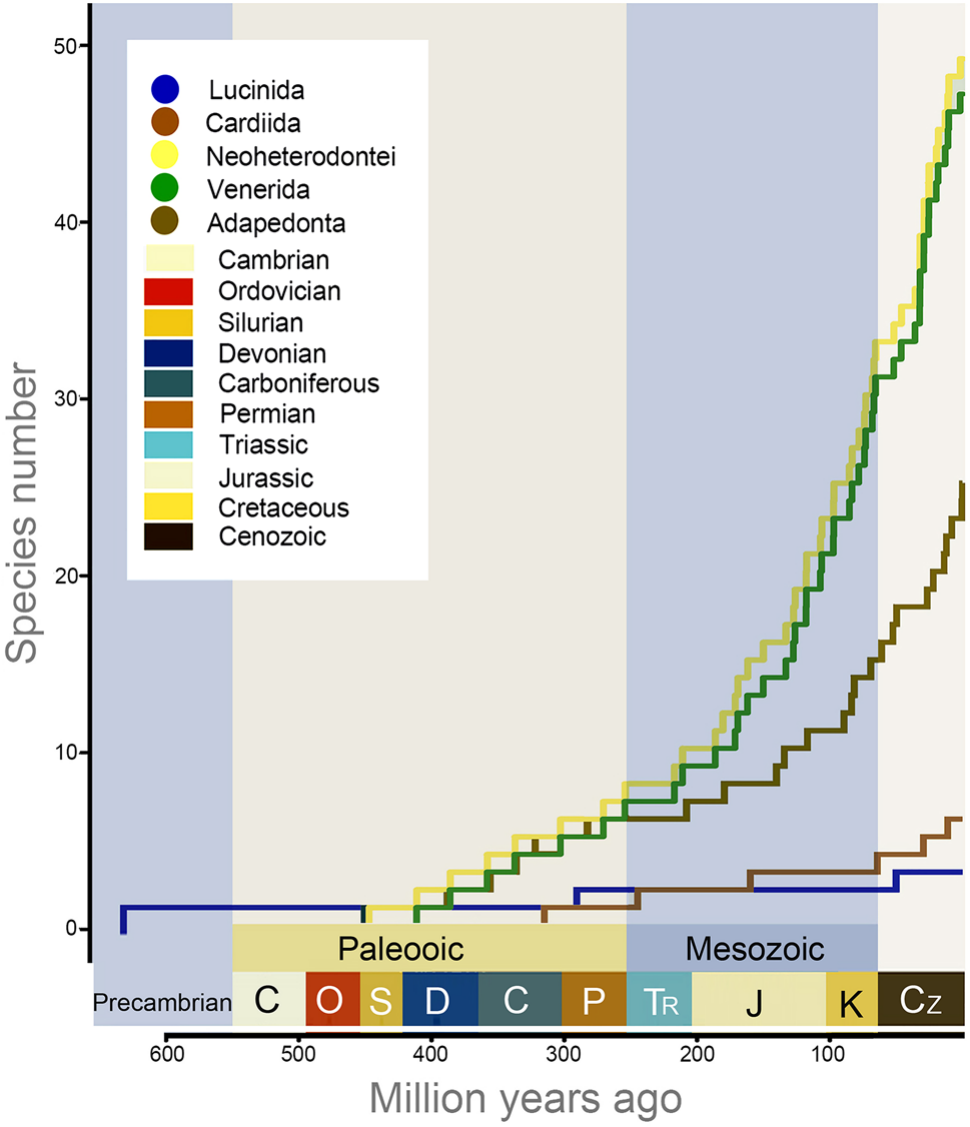
Lineage through time trajectories for Imparidentia taxa

The appearance of *Cypricardites bidens* in the fossil record clearly points out an early origin of Imparidentia bivalves before the Ordovician (Isberg 1934). The divergence of this clade was dated to the Precambrian (Ediacaran Period) with molecular techniques, although no undisputable fossil evidence for Imparidentia has ever been found in sediments older than the earliest Cambrian. Molecular divergence time estimation suggests that the superorder Imparidentia diversified steadily since its origin and continued generating higher diversity until the Cretaceous, seemingly unaffected by the Permian mass extinction event (Fig. 4). The long branches separating its origin and diversification from modern forms are likely indicative of major-lineage extinction.

The divergence time estimation for Lucinida has shown this group originated during the Ediacaran and first diversified before the end-Permian mass extinction. However, the fossil record of Lucinida could reach the early Silurian, about 443.7 Ma, exemplified by *Illionia canadensi* from Estonia, Sweden and the USA (Michigan). Generally, the fossil record for many invertebrate groups is scarce (Ballesteros et al. 2020). Cardiida was represented by *Cypricardites bidens* from the Early Silurian onwards (Isberg 1934). The first diversification of Cardiida during the Ordovician as shown in time trees is concordant with the fossil record, and further diversification occurred steadily until the Cretaceous.

Our estimation of divergence time undoubtedly indicated Cambrian to Ordovician ages of Neoheterodontei origin. The timing of the split between Adapedonta and remaining Neoheterodontei bivalves was estimated to have occurred during the Ordovician in our time-calibrated phylogenetic analysis. Adapedonta first became abundant during the Triassic after the end-Permian event. The divergence of Myida from Venerida is dated to the Ordovician Period (447 Ma) using molecular techniques in our analysis. Clavate boring in wood (lignic) substrates has been attributed to bivalve trace-makers (Teredinidae and Pholadidae) from Middle Jurassic to the present day (Bromley 2004; Evans 1999). However, a trace fossil bored by bioeroding bivalves could extend to the Ordovician (Taylor and Wilson 2003). These borers are morphologically indistinguishable, usually being differentiated by substrate or the mode of the boring process (Donovan and Ewin 2018). Thus, these putative ichnospecies may be the ancestor of Myida, although this needs further verification. The fossil record of Venerida dates to the Late Ordovician in the form of *Glauconome plumula* (Skjeseth 1955) which is consistent with our molecular estimation*.* Despite these relatively deep origins, fossil record of Venerida was scarce throughout the period spanning the Silurian to the Carboniferous. The rare fossil record extending to the Paleozoic is inconclusive for determining whether the diversification of this group was ancient. Evolutionary relicts like Nautiloidea appeared early in the fossil record, the oldest fossils belonging to the Late Cambrian, but this lineage has survived to the present day as a small number of species that diverged recently (Combosch et al. 2017b). However, patterns of Venerida species richness observed in our analysis dates the beginning of the rapid radiation of venerids to the middle of Devonian conflicts with the fossil record.

The findings of the present study support the overall backbone of the Imparidentia phylogeny. The monophyly of Imparidentia and four main lineages—Cardiida, Adapedonta, Myida and Venerida—was considered valid, although the monophyly of Lucinida remains uncertain. Our results strongly support Thyasiridae as the sister group to a clade that contains the rest of Imparidentia except Lucinida. Chamidae formed a clade with Mactridae as a member of the Venerida. Moreover, molecular divergence times were inferred by using extinct taxa to calibrate nine nodes in the Imparidentia tree. The origin of these major clades ranged from the Ordovician to the Permian with diversification through the Palaeozoic and Mesozoic. However, we lack sufficiently broad geographic sampling of Imparidentia to reconstruct a more complete phylogenetic tree. Denser sampling of extant Imparidentia bivalve diversity in future studies will augment the predictive power of such approaches in deciphering the evolutionary history of this group.

## Materials and methods

### DNA extraction, library preparation, and next-generation sequencing

The newly sequenced specimens were obtained from the intertidal zones along the South China Sea coast in Hainan, China. Voucher specimens of *C. asperella*, *C. limbula*, *C. dunkeri*, *B. manilensis* and *C. divergens* are preserved in Key Laboratory of Mariculture, Ocean University of China. Genomic DNA (gDNA) was obtained from tissues preserved in ethanol using a modified phenol–chloroform procedure (Li et al. 2002). Total genomic DNA was quantified using a Nanodrop spectrophotometer and then supplied to Novogene (Beijing, China) for library preparation and high-throughput sequencing. Libraries were prepared with an average fragment size of approximately 300 bp. The DNA libraries were sequenced on the Illumina HiSeq 4000 platform using parallel sequencing with 150 bp paired-end reads. The raw data produced for each library were filtered for further analysis. De novo assemblies of reads and annotation were conducted with Mitoz using the invertebrate genetic code. In the case of *C. limbula* and *C. dunkeri*, the largest mt genomes contig generated by Mitoz lacking several mt genes and Geneious Prime version 2020.1 (Hahn et al. 2013) was used for assembly.

### Gene annotation

The protein-coding genes (PCGs) predictions were mainly accomplished using the Open Reading Frame Finder and MITOS web server (Bernt et al. 2013) with the invertebrate mitochondrial genetic code. Gene boundaries were determined by comparison with the mitochondrial genome download from NCBI. The rRNA genes were recognized by BLAST search (http://www.ncbi.nlm.nih.gov/BLAST/) employing their resemblance of inferred sequences and presumed to stretch out the boundaries of nearby genes (Boore et al. 2005). ARWEN (Laslett and Canbäck 2008) and DOGMA (Wyman et al. 2004) were used to determine the positions of tRNA genes using the invertebrate mitochondrial genetic code and the default search mode.

### Sequence analysis and principal component analysis

Principal component analysis (PCA) was carried out with numerous diverse variables (Supplementary Table S1) including length of mt genome, percentage of major Unassigned Regions, A-T and G-C skews, AT content, the total number of genes, and mitochondrial gene rearrangements. The rRNAs and tRNAs were eliminated from the analysis due to many uncertainties in annotations, therefore the rearrangements rely solely on PCGs. The rates of mitochondrial rearrangement of the orders were quantified using the Amount of Mitochondrial Identical Gene Arrangements (AMIGA) according to Plazzi et al. (2016). This was given by AMIGA = (N_IGA_–1)/(N-1) where N_IGA_ is the number of taxa in the analyzed sample that share an Identical Gene Arrangement and N is the total number of taxa. PCA was conducted in the R environment. MEGA 7 was used to calculate nucleotide compositions and A + T content values of mitochondrial genomes (Kumar et al. 2016). The dN/dS (ω) was determined for each PCG using codeml of PAML v. 4.8 (Yang 2007) along the phylogenetic tree (Supplementary Fig. S2) (dN is the ratio of non-synonymous substitutions and dS is the ratio of synonymous substitutions). Due to the poor sample size of Myida (*N* = 2), this order was excluded from dN/dS analyses. We performed a chi-square distribution with default settings to compare the lnL of null hypothesis and alternative hypothesis of dN/dS (*ω*) for each PCG in the R environment (Wong et al. 2004).

### Phylogenetic analyses

Amino acid sequences for the 12 mitochondrial PCGs were produced from new sequences of mitochondrial genomes, as well as 81 Imparidentia mitochondrial genomes available on NCBI (see Supplementary Table S1). Given the many uncertainties in annotation and high rate of evolution, *atp*8 gene, rRNA and tRNAs were discarded from the phylogenetic analyses. *Laternula elliptica* (order Anomalodesmata) and *Perna viridis* (order Mytilida) were selected as outgroup taxa in accordance with two recent phylogenomic studies (Plazzi et al. 2016; Williams et al. 2017). Alignments of single PCGs were produced for amino acid sequences with MAFFT (Katoh et al. 2005). Alignments were trimmed with Gbloks v.0.91b (Cruickshank 2000) cull overhanging ends. The concatenated amino acid supermatrix was created with Sequence Matrix 1.7.8 (Vaidya et al. 2011). The best-fit partition schemes and molecular evolution models were forecast using PartitionFinder 2.1.0 (PF2) based on the BIC approach. This analysis identified genes grouped by enzymatic complexes (atp, cox, cob, nad) as the best-fit partitions and the best-fit substitution model as shown in Supplementary Table S3.

The software IQ-TREE v.1.6.8 (Nguyen et al. 2015) was applied to infer ML phylogeny. The maximum likelihood tree was constructed with 10,000 ultrafast bootstrap replicates. Bayesian inference analysis was conducted using MrBayes 3.2.6 (Huelsenbeck and Ronquist 2001) with four parallel Markov chain Monte Carlo (MCMC) chains for 10^8^ generations, sampling every 1000 generations and discarding the first 2,500,000 generations as burn-in. Above parameters were assessed with Tracer v. 1.7 (Rambaut et al. 2018).

### Divergence times estimation

BEAST v.1.7 (Drummond et al. 2012) implementing the model of the Yule process was utilized to estimate the divergence times among clades using the amino acid sequences of 12 PCGs. The tree topology and node ages were estimated using an uncorrelated lognormal clock model and birth–death model was set as the tree prior. Two independent Markov chains were run for 100 million generations, with sampling every 10^4^ generations. The two independent runs were then combined, after discarding the first 10% generations as burn-in, using TreeAnnotator 1.10.4. The convergence of chains was visually inspected in Tracer v. 1.7.1. The ESS values of most of the parameters were greater than 200. MCC trees were produced in TreeAnnotator v. 1.10.4 and visualized in FigTree v.1.4.3 (Rambaut 2014).

Nine node calibrations were used to estimate divergence times. Available data in Paleobiology Database (https://paleobiodb.org/) were used to incorporate fossil ages. The oldest fossil assigned to the branch of Cardiidae is the fossil record of Pleurophoridae from the Upper Ordovician (445.6–455.8 Mya) which was used to calibrate the divergence of this group (Isberg 1934). The divergence of Tellinoidea was constrained between 326.4 and 318.1 Mya (based on the fossil record of *Solecurtus*) (Elias 1957). The oldest fossil of Hiatellidae, namely *Panopea anabarica*, dates to the Triassic (242–247.2 Mya) (Konstantinov et al. 2007). The root age of Pholadidae based on the fossil of *Martesia* sp*.* was constrained spanning 189.6 to 183 Mya (Velazco 2008). For Veneridae, we constrained the basal diversification of this group between 339.4–336 Mya, based on the fossil *Pullastra striatocostata* (Coy 1847). The divergence of Cyrenidae was constrained between 189.6 and 183 Mya on the basis of *Cyrena* sp. (Bouchet et al. 2010). The branch age of *Paphia* was restricted to at least 112.6 Mya (age of *Paphia peruana*) (Kummel 1948). The divergence of *Dosinia* was set to 97 Mya, based on the age of *Dosinia delettrei* (99.7–94.3 Mya) (Perrilliat et al. 2006). The outgroup taxa were constrained to between 478.6–466 Mya based on the age of *Dipleurodonta* sp*.* (Vaccari and Waisfeld 1994).

The lineage through time plots (LTT) was conducted by the R package ape v. 5.0 (Paradis and Schliep 2019) based on the phylogentic tree to explore the diversification rates through time.

## Supplementary Information

Below is the link to the electronic supplementary material.

Supplementary file1 (DOCX 1218 KB)

## Data Availability

The raw reads used for mtDNA genome assemblies (MZ701706, MZ557447, MZ540208, MZ701705, MZ688407) have been deposited at Short Read Archive (SRA) at the National center for Biotechnology Information (NCBI) under the accession SUB11188349.
