# Supplementary material for: Phylogenomic resolution of Imparidentia (Mollusca: Bivalvia) diversification through mitochondrial genomes
Source: Mar Life Sci Technol. 2023 Jun 19;5(3):326–36. doi: 10.1007/s42995-023-00178-x (PMC10449738; doi:10.1007/s42995-023-00178-x)
Supplement: Supplementary file 1 — Supplementary file1 (DOCX 1218 KB) [file 42995_2023_178_MOESM1_ESM.docx]

Table S1. Complete mitochondrial genomes and main features used for phylogenetic analysis in this study.

| Species | Order | Superfamily | Family | Accession number | Length  (bp) | UR% | Gene  number | A+T | G+C | AT  skew | GC  skew | AMIGA |
| --- | --- | --- | --- | --- | --- | --- | --- | --- | --- | --- | --- | --- |
| *Solen strictus* | Adapedonta | Solenoidea | Solenidae | NC017616 | 16535 | 8.13 | 36 | 0.63 | 0.37 | -0.31 | 0.37 | 0.33 |
| *Solen grandis* | Adapedonta | Solenoidea | Solenidae | NC016665 | 16784 | 9.66 | 36 | 0.65 | 0.35 | -0.30 | 0.39 | 0.33 |
| *Panopea globosa* | Adapedonta | Hiatelloidea | Hiatellidae | NC025636 | 15469 | 5.10 | 37 | 0.64 | 0.36 | -0.27 | 0.44 | 0.33 |
| *Sinonovacula constricta* | Adapedonta | Tellinoidea | Solecurtidae | NC011075 | 17225 | 13.47 | 33 | 0.67 | 0.33 | -0.23 | 0.36 | 0.33 |
| *Panopea generosa* | Adapedonta | Hiatelloidea | Hiatellidae | NC025635 | 15585 | 5.92 | 37 | 0.64 | 0.36 | -0.22 | 0.38 | 0.33 |
| *Panopea abrupta* | Adapedonta | Hiatelloidea | Hiatellidae | NC033538 | 15381 | 5.89 | 37 | 0.64 | 0.36 | -0.20 | 0.37 | 0.33 |
| *Hiatella arctica* | Adapedonta | Hiatelloidea | Hiatellidae | NC008451 | 18244 | 13.63 | 38 | 0.66 | 0.34 | -0.15 | 0.29 | 0.33 |
| *Solecurtus divaricatus* | Cardiida | Tellinoidea | Solecurtidae | NC018376 | 16749 | 6.90 | 36 | 0.60 | 0.40 | -0.29 | 0.38 | 0.56 |
| *Sanguinolaria ovalis* | Cardiida | Tellinoidea | Psammobiidae | NC042423 | 16460 | 6.61 | 36 | 0.61 | 0.39 | -0.27 | 0.36 | 0.56 |
| *Hiatula diphos* | Cardiida | Tellinoidea | Psammobiidae | NC018372 | 16352 | 5.50 | 36 | 0.63 | 0.37 | -0.26 | 0.37 | 0.56 |
| *Hiatula acuta* | Cardiida | Tellinoidea | Psammobiidae | NC042421 | 16352 | 5.35 | 37 | 0.63 | 0.37 | -0.26 | 0.37 | 0.56 |
| *Hiatula chinensis* | Cardiida | Tellinoidea | Psammobiidae | NC042420 | 16333 | 5.72 | 36 | 0.61 | 0.39 | -0.25 | 0.32 | 0.56 |
| *Gari elongata* | Cardiida | Tellinoidea | Psammobiidae | NC042422 | 16766 | 8.68 | 36 | 0.62 | 0.38 | -0.23 | 0.35 | 0.56 |
| *Semele scabra* | Cardiida | Tellinoidea | Semelidae | NC018374 | 17117 | 9.13 | 35 | 0.59 | 0.41 | -0.23 | 0.43 | 0.56 |
| *Iridona iridescens* | Cardiida | Tellinoidea | Tellinidae | NC018371 | 16799 | 9.53 | 36 | 0.66 | 0.34 | -0.22 | 0.35 | 0.56 |
| *Semelidae gen. et sp.* | Cardiida | Tellinoidea | Semelidae | KX815956 | 16270 | 6.63 | 37 | 0.68 | 0.32 | -0.20 | 0.26 | 0.56 |
| *Scrobicularia plana* | Cardiida | Tellinoidea | Scrobiculariidae | NC046518 | 16170 | 7.41 | 37 | 0.67 | 0.33 | -0.18 | 0.26 | 0.56 |
| *Acanthocardia tuberculata* | Cardiida | Cardioidea | Cardiidae | NC008452 | 16104 | 10.17 | 37 | 0.60 | 0.40 | -0.18 | 0.17 | 0.56 |
| *Macoma balthica* | Cardiida | Tellinoidea | Tellinidae | NC046519 | 17492 | 14.18 | 37 | 0.64 | 0.37 | -0.18 | 0.29 | 0.56 |
| *Donax variegatus* | Cardiida | Tellinoidea | Donacidae | NC035986 | 17195 | 10.39 | 37 | 0.60 | 0.40 | -0.18 | 0.21 | 0.56 |
| *Cerastoderma edule* | Cardiida | Cardioidea | Cardiidae | NC035728 | 14947 | 2.55 | 39 | 0.58 | 0.42 | -0.18 | 0.15 | 0.56 |
| *Tridacna crocea* | Cardiida | Cardioidea | Tridacnidae | MK249738 | 19157 | 19.67 | 39 | 0.62 | 0.38 | -0.16 | 0.21 | 0.56 |
| *Nuttallia obscurata* | Cardiida | Tellinoidea | Psammobiidae | NC018373 | 18182 | 14.59 | 36 | 0.65 | 0.35 | -0.15 | 0.32 | 0.56 |
| *Donax trunculus* | Cardiida | Tellinoidea | Donacidae | NC035985 | 17365 | 10.78 | 37 | 0.59 | 0.41 | -0.14 | 0.21 | 0.56 |
| *Tridacna noae* | Cardiida | Cardioidea | Tridacnidae | MT755624 | 20548 | 25.75 | 39 | 0.62 | 0.38 | -0.14 | 0.24 | 0.56 |
| *Fulvia mutica* | Cardiida | Cardioidea | Cardiidae | NC022194 | 19110 | 21.91 | 42 | 0.65 | 0.36 | -0.13 | 0.28 | 0.56 |
| *Donax semistriatus* | Cardiida | Tellinoidea | Donacidae | NC035984 | 17044 | 9.28 | 37 | 0.62 | 0.38 | -0.13 | 0.18 | 0.56 |
| *Tridacna derasa* | Cardiida | Cardioidea | Tridacnidae | NC039945 | 20760 | 24.26 | 38 | 0.65 | 0.35 | -0.13 | 0.27 | 0.56 |
| *Tridacna squamosa* | Cardiida | Cardioidea | Tridacnidae | NC026558 | 20930 | 27.32 | 38 | 0.62 | 0.38 | -0.12 | 0.19 | 0.56 |
| *Hippopus hippopus* | Cardiida | Cardioidea | Tridacnidae | MG722975 | 22463 | 28.93 | 38 | 0.60 | 0.40 | -0.12 | 0.23 | 0.56 |
| *Tridacna gigas* | Cardiida | Cardioidea | Tridacnidae | NC050683 | 19558 | 23.07 | 38 | 0.58 | 0.42 | -0.11 | 0.21 | 0.56 |
| *Donax vittatus* | Cardiida | Tellinoidea | Donacidae | NC035987 | 17070 | 9.31 | 37 | 0.64 | 0.36 | -0.11 | 0.17 | 0.56 |
| *Lutraria maxima* | Venerida | Mactroidea | Mactridae | NC036766 | 17082 | 14.31 | 37 | 0.64 | 0.36 | -0.29 | 0.39 | 0.26 |
| *Periglypta puerpera* | Venerida | Veneroidea | Veneridae | MT737377 | 16855 | 8.91 | 39 | 0.65 | 0.35 | -0.29 | 0.52 | 0.26 |
| *Lutraria rhynchaena* | Venerida | Mactroidea | Mactridae | NC023384 | 16927 | 7.38 | 36 | 0.62 | 0.38 | -0.28 | 0.40 | 0.26 |
| *Cyclina sinensis* | Venerida | Veneroidea | Veneridae | KU097333 | 21799 | 25.57 | 37 | 0.73 | 0.27 | -0.28 | 0.42 | 0.26 |
| *Meretrix lamarckii* | Venerida | Veneroidea | Veneridae | NC016174 | 21209 | 22.03 | 37 | 0.66 | 0.34 | -0.28 | 0.43 | 0.26 |
| *Meretrix lyrata* | Venerida | Veneroidea | Veneridae | NC022924 | 21625 | 24.23 | 41 | 0.71 | 0.30 | -0.27 | 0.45 | 0.26 |
| *Meretrix lusoria* | Venerida | Veneroidea | Veneridae | NC014809 | 20268 | 20.04 | 37 | 0.68 | 0.32 | -0.26 | 0.42 | 0.26 |
| *Villorita cyprinoides* | Venerida | Cyrenoidea | Cyrenidae | NC050989 | 15880 | 3.43 | 37 | 0.68 | 0.32 | -0.26 | 0.43 | 0.26 |
| *Meretrix petechialis* | Venerida | Veneroidea | Veneridae | NC012767 | 19567 | 16.68 | 36 | 0.68 | 0.32 | -0.26 | 0.39 | 0.26 |
| *Meretrix meretrix* | Venerida | Veneroidea | Veneridae | NC013188 | 19826 | 17.42 | 37 | 0.68 | 0.32 | -0.25 | 0.39 | 0.26 |
| *Placamen foliaceum* | Venerida | Veneroidea | Veneridae | MT737375 | 17789 | 12.93 | 40 | 0.69 | 0.31 | -0.25 | 0.43 | 0.26 |
| *Gafrarium pectinatum* | Venerida | Veneroidea | Veneridae | MT737369 | 21132 | 19.69 | 38 | 0.66 | 0.34 | -0.24 | 0.44 | 0.26 |
| *Calyptogena extenta* | Venerida | Glossoidea | Vesicomyidae | MF981085 | 16106 | 6.8 | 37 | 0.66 | 0.35 | -0.23 | 0.40 | 0.26 |
| *Corbicula fluminea* | Venerida | Corbiculoidea | Corbiculidae | NC046410 | 17423 | 13.27 | 37 | 0.71 | 0.30 | -0.23 | 0.40 | 0.26 |
| *Placamen isabellina* | Venerida | Veneroidea | Veneridae | MT737376 | 18602 | 18.17 | 38 | 0.69 | 0.31 | -0.23 | 0.37 | 0.26 |
| *Mactra chinensis* | Venerida | Mactroidea | Mactridae | NC025510 | 17285 | 12.94 | 35 | 0.64 | 0.36 | -0.22 | 0.26 | 0.26 |
| *Pliocardia ponderosa* | Venerida | Glossoidea | Vesicomyidae | MF981084 | 16275 | 7.3 | 37 | 0.68 | 0.32 | -0.22 | 0.42 | 0.26 |
| *Saxidomus purpurata* | Venerida | Veneroidea | Veneridae | NC026728 | 19637 | 20.76 | 36 | 0.66 | 0.34 | -0.22 | 0.35 | 0.26 |
| *Mercenaria mercenaria* | Venerida | Veneroidea | Veneridae | NC048487 | 18365 | 19.28 | 37 | 0.69 | 0.31 | -0.21 | 0.39 | 0.26 |
| *Calyptogena marissinica* | Venerida | Glossoidea | Vesicomyidae | NC044766 | 17374 | 13.35 | 37 | 0.65 | 0.35 | -0.21 | 0.38 | 0.26 |
| *Antigona lamellaris* | Venerida | Veneroidea | Veneridae | MT737368 | 18209 | 15.59 | 38 | 0.68 | 0.32 | -0.21 | 0.41 | 0.26 |
| *Circe scripta* | Venerida | Veneroidea | Veneridae | MT737370 | 20182 | 17.59 | 38 | 0.73 | 0.27 | -0.20 | 0.42 | 0.26 |
| *Dosinia altior* | Venerida | Veneroidea | Veneridae | NC037916 | 17536 | 13.73 | 37 | 0.70 | 0.30 | -0.20 | 0.43 | 0.26 |
| *Mactra antiquata* | Venerida | Mactroidea | Mactridae | NC021375 | 17199 | 10.94 | 36 | 0.64 | 0.36 | -0.20 | 0.30 | 0.26 |
| *Abyssogena phaseoliformis* | Venerida | Glossoidea | Vesicomyidae | AP014557 | 19424 | 21.4 | 39 | 0.70 | 0.30 | -0.20 | 0.44 | 0.26 |
| *Turneroconcha magnifica* | Venerida | Glossoidea | Vesicomyidae | NC028724 | 19738 | 22.36 | 36 | 0.68 | 0.32 | -0.20 | 0.39 | 0.26 |
| *Dosinia japonica* | Venerida | Veneroidea | Veneridae | NC038063 | 17693 | 15.27 | 37 | 0.70 | 0.30 | -0.19 | 0.39 | 0.26 |
| *Dosinia troscheli* | Venerida | Veneroidea | Veneridae | NC037917 | 17229 | 12.58 | 37 | 0.70 | 0.30 | -0.19 | 0.41 | 0.26 |
| *Callista erycina* | Venerida | Veneroidea | Veneridae | MT737374 | 20353 | 19.15 | 38 | 0.67 | 0.33 | -0.19 | 0.33 | 0.26 |
| *Pseudocardium sachalinense* | Venerida | Mactroidea | Mactridae | MG431821 | 17978 | 11.79 | 37 | 0.59 | 0.41 | -0.18 | 0.23 | 0.26 |
| *Arctica islandica* | Venerida | Arcticoidea | Arcticidae | NC022709 | 18289 | 16.2 | 36 | 0.69 | 0.31 | -0.16 | 0.30 | 0.26 |
| *Tapes conspersus* | Venerida | Veneroidea | Veneridae | MT737378 | 19048 | 14.45 | 38 | 0.65 | 0.35 | -0.16 | 0.29 | 0.26 |
| *Leukoma jedoensis* | Venerida | Veneroidea | Veneridae | MT737372 | 18847 | 14.7 | 38 | 0.69 | 0.31 | -0.16 | 0.31 | 0.26 |
| *Macridiscus multifarius* | Venerida | Veneroidea | Veneridae | NC045888 | 20171 | 21.72 | 36 | 0.68 | 0.32 | -0.16 | 0.35 | 0.26 |
| *Paratapes undulatus* | Venerida | Veneroidea | Veneridae | NC016891 | 18154 | 11.99 | 38 | 0.65 | 0.35 | -0.15 | 0.36 | 0.26 |
| *Paphia euglypta* | Venerida | Veneroidea | Veneridae | NC014579 | 18643 | 13.65 | 37 | 0.67 | 0.33 | -0.15 | 0.35 | 0.26 |
| *Paratapes textilis* | Venerida | Veneroidea | Veneridae | NC016890 | 18561 | 13.42 | 38 | 0.64 | 0.36 | -0.14 | 0.37 | 0.26 |
| *Anomalodiscus squamosus* | Venerida | Veneroidea | Veneridae | MT737366 | 17699 | 13.71 | 40 | 0.70 | 0.30 | -0.14 | 0.32 | 0.26 |
| *Tapes belcheri* | Venerida | Veneroidea | Veneridae | MT737367 | 18393 | 13.3 | 38 | 0.68 | 0.32 | -0.14 | 0.34 | 0.26 |
| *Ruditapes philippinarum* | Venerida | Veneroidea | Veneridae | NC031332 | 22089 | 16.51 | 39 | 0.69 | 0.30 | -0.14 | 0.36 | 0.26 |
| *Marcia hiantina* | Venerida | Veneroidea | Veneridae | MT737373 | 20924 | 22.62 | 39 | 0.68 | 0.32 | -0.14 | 0.30 | 0.26 |
| *Macridiscus aequilatera* | Venerida | Veneroidea | Veneridae | NC045870 | 20738 | 25.62 | 36 | 0.67 | 0.33 | -0.14 | 0.29 | 0.26 |
| *Ruditapes decussatus* | Venerida | Veneroidea | Veneridae | NC035757 | 18995 | 14.12 | 37 | 0.63 | 0.37 | -0.11 | 0.28 | 0.26 |
| *Paphia amabilis* | Venerida | Veneroidea | Veneridae | NC016889 | 19629 | 17.32 | 38 | 0.63 | 0.37 | -0.11 | 0.29 | 0.26 |
| *Cryptonema producta* | Venerida | Veneroidea | Veneridae | MT737371 | 20423 | 19.13 | 39 | 0.70 | 0.30 | -0.10 | 0.32 | 0.26 |
| *Chama asperella** | Venerida | Chamoidea | Chamidae | MZ701706 | 26915 | 39.6 | 37 | 0.58 | 0.42 | -0.25 | 0.45 | 0.26 |
| *Chama dunkeri** | Venerida | Chamoidea | Chamidae | MZ557447 | 22537 | 18.9 | 39 | 0.59 | 0.41 | -0.23 | 0.41 | 0.26 |
| *Chama limbula** | Venerida | Chamoidea | Chamidae | MZ688407 | 20179 | 15.6 | 37 | 0.59 | 0.41 | -0.24 | 0.41 | 0.26 |
| *Lucinella divaricata* | Lucinida | Lucinoidea | Lucinidae | NC013275 | 18940 |  |  |  |  |  |  |  |
| *Loripes orbiculatus* | Lucinida | Lucinoidea | Lucinidae | NC013271 | 17321 |  |  |  |  |  |  |  |
| *Conchocele cf. bisecta* | Lucinida | Thyasiroidea | Thyasiridae | LC126312 | 17188 |  |  |  |  |  |  |  |
| *Ctena divergens** | Lucinida | Lucinoidea | Lucinidae | MZ540208 | 23414 |  |  |  |  |  |  |  |
| *Mya arenaria* | Myida | Myoidea | Myidae | NC024738 | 17947 |  |  |  |  |  |  |  |
| *Barnea manilensis** | Myida | Pholadoidea | Pholadidae | MZ701705 | 20326 |  |  |  |  |  |  |  |

Note: The newly sequenced complete mt genomes are indicated with an asterisk (*). Lucinida and Myida were not included in PCA.

Table S2. The dN/dS ratio for 12 protein coding genes of Imparidentia bivalves.

| gene | Imparidentia | Lucinida | Cardiida | Adapedonta | Venerida |
| --- | --- | --- | --- | --- | --- |
| *atp6* | 0.0343 | 0.08803 | 0.02315 | 0.03983 | 0.03945 |
| *cob* | 0.03372 | 0.02192 | 0.02425 | 0.03609 | 0.03919 |
| *cox1* | 0.02576 | 0.03574 | 0.01644 | 0.01665 | 0.03135 |
| *cox2* | 0.03674 | 0.02397 | 0.0242 | 0.03285 | 0.05041 |
| *cox3* | 0.02249 | 0.00769 | 0.01595 | 0.008 | 0.02968 |
| *nad1* | 0.0392 | 0.07231 | 0.02954 | 0.03228 | 0.04367 |
| *nad2* | 0.06129 | 0.06549 | 0.06574 | 0.04804 | 0.05868 |
| *nad3* | 0.02302 | 0.03463 | 0.01699 | 0.02518 | 0.02448 |
| *nad4* | 0.0483 | 0.06033 | 0.04552 | 0.03218 | 0.05007 |
| *nad5* | 0.05541 | 0.05425 | 0.04849 | 0.03063 | 0.0637 |
| *nad6* | 0.04671 | 0.05302 | 0.03214 | 0.05534 | 0.05566 |
| *nad4L* | 0.05102 | 0.0445 | 0.0351 | 0.03202 | 0.06452 |

Table S3. The best fit substitution model of amino acid data set.

|  | Partitions | Best Model |
| --- | --- | --- |
| 1 | *atp6* | JTT+G+F |
| 2 | *cox1-2-3* | LG+I+G+F |
| 3 | *cob* | LG+I+G+F |
| 4 | *nad1-2-3-4-4L-5-6* | MTART+I+G+F |


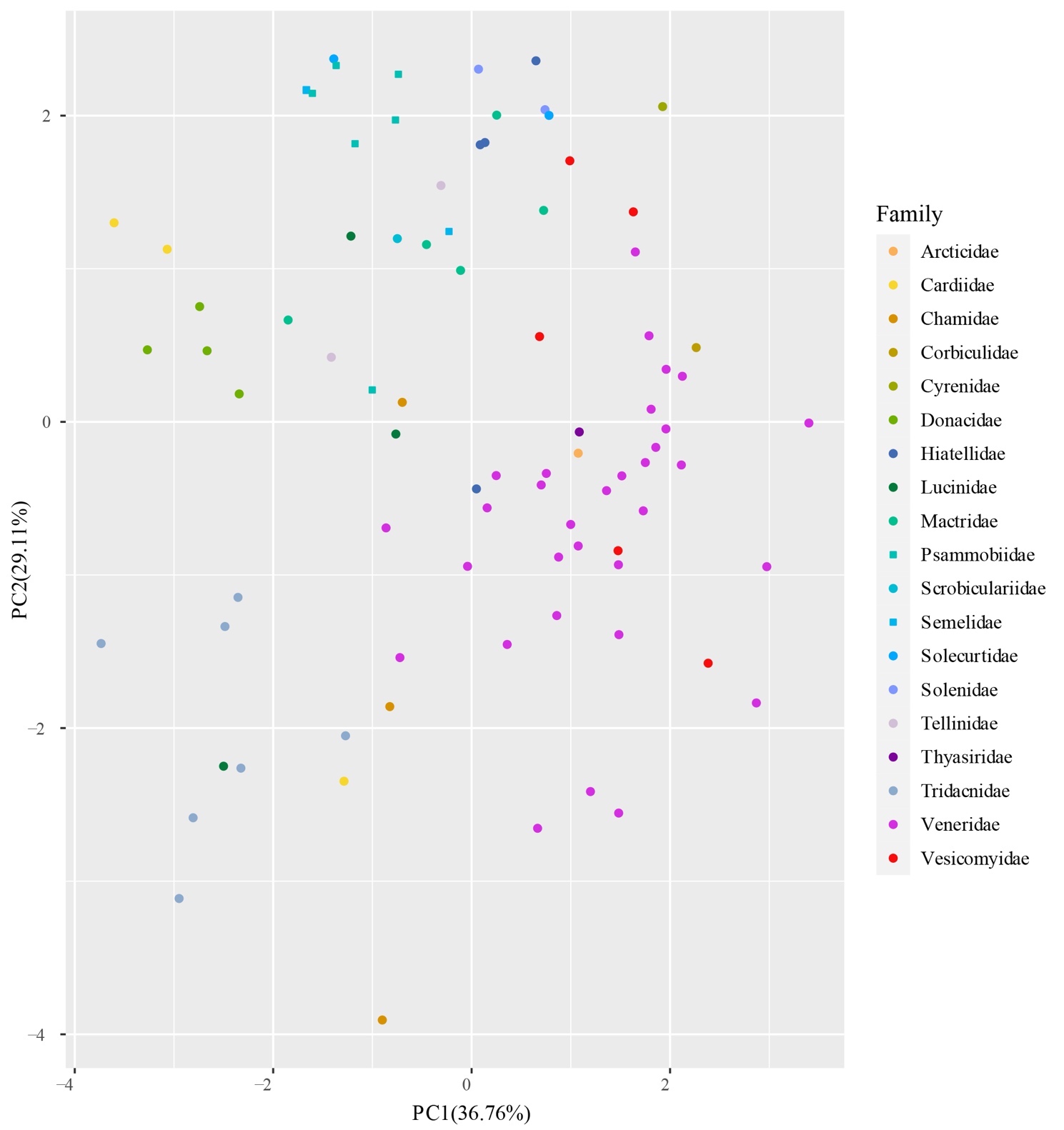


Fig. S1 Imparidentia mitogenomics PCA. Different families are indicated with different colors. PC, principal component.

Fig. S2 Maximum-likelihood tree of Imparidentia bivalves based on the amid animo sequences of 12 mitochondrial protein-coding genes. Bootstrap support values are presented at each node. The differences between ML tree and BI tree are shown in red frames.


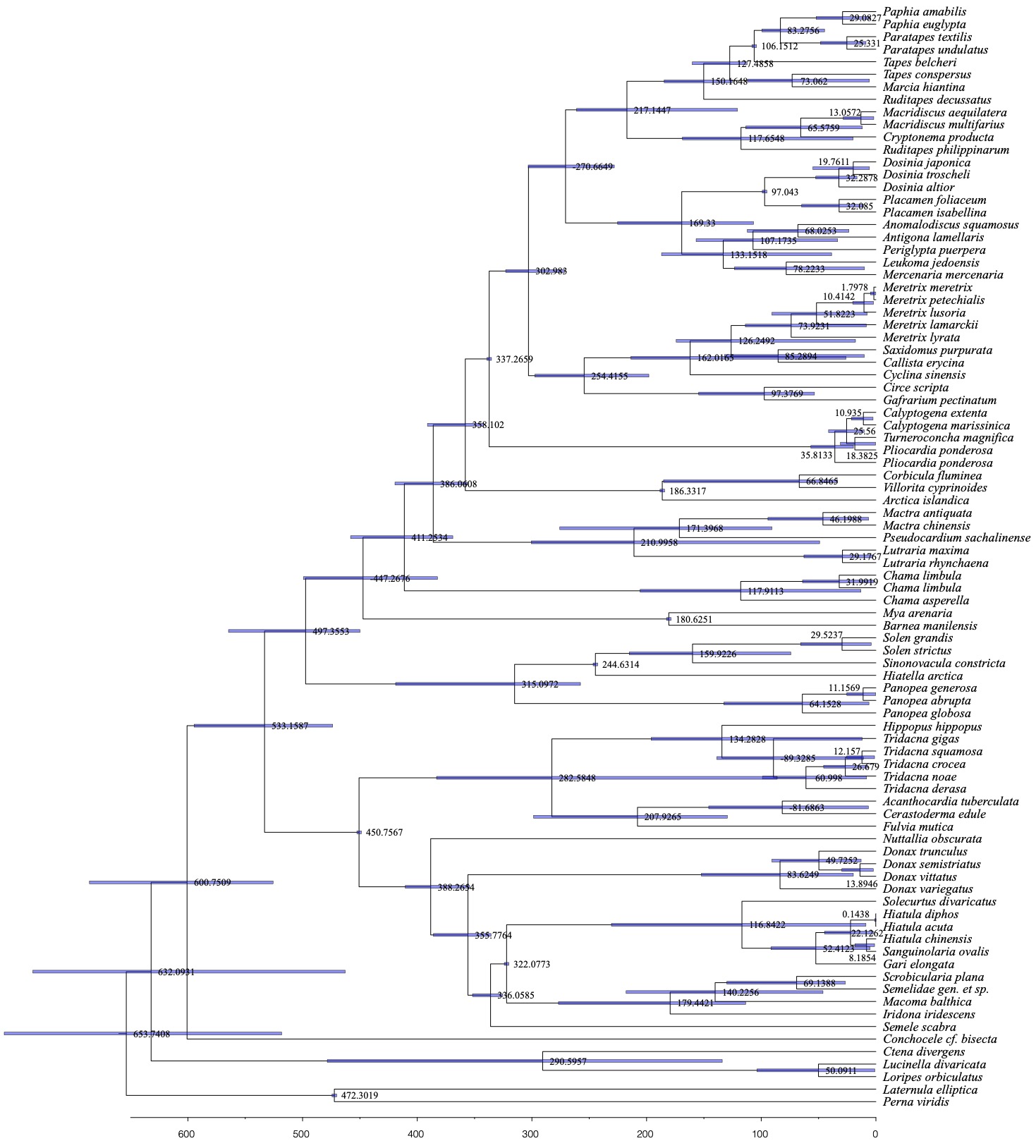


Fig. S3 Divergence time estimation analysis of Imparidentia inferred via Bayesian inference under the uncorrelated relaxed lognormal clock model. Dates (and credibility intervals) are in millions of years, and horizontal bars represent 95% credibility intervals of relevant nodes.
